# What drives sound symbolism? Different acoustic cues underlie sound-size and sound-shape mappings

**DOI:** 10.1038/s41598-017-05965-y

**Published:** 2017-07-17

**Authors:** Klemens Knoeferle, Jixing Li, Emanuela Maggioni, Charles Spence

**Affiliations:** 1 Center for Multisensory Marketing, Department of Marketing, BI Norwegian Business School, Oslo, Norway; 2 Department of Linguistics, Cornell University, Ithaca, United States; 3 SCHI Lab, Creative Technology Research Group, School of Engineering and Informatics, University of Sussex, Brighton, United Kingdom; 4 Department of Experimental Psychology, University of Oxford, Oxford, United Kingdom

## Abstract

Sound symbolism refers to the non-arbitrary mappings that exist between phonetic properties of speech sounds and their meaning. Despite there being an extensive literature on the topic, the acoustic features and psychological mechanisms that give rise to sound symbolism are not, as yet, altogether clear. The present study was designed to investigate whether different sets of acoustic cues predict size and shape symbolism, respectively. In two experiments, participants judged whether a given consonant-vowel speech sound was large or small, round or angular, using a size or shape scale. Visual size judgments were predicted by vowel formant F1 in combination with F2, and by vowel duration. Visual shape judgments were, however, predicted by formants F2 and F3. Size and shape symbolism were thus not induced by a common mechanism, but rather were distinctly affected by acoustic properties of speech sounds. These findings portray sound symbolism as a process that is not based merely on broad categorical contrasts, such as round/unround and front/back vowels. Rather, individuals seem to base their sound-symbolic judgments on specific sets of acoustic cues, extracted from speech sounds, which vary across judgment dimensions.

## Introduction

Sound (or phonetic) symbolism refers to the non-arbitrary mappings that exist between phonetic properties of labels and perceptual properties of their referents. In a classic early study of sound-size associations, 74.6–96.4% of the 500 participants tested across a number of experiments associated non-words containing the vowel “a” (e.g., as in “mal”) with larger objects, and non-words containing the vowel “i” (e.g., “mil”) with smaller objects^1^. In another classic study on sound-shape associations, participants systematically mapped a curvy cloud-like shape with the word “baluba”, and an angular star-like shape with “takete”^2^. Building on these pioneering experiments, both more subtle behavioural studies^3, 4^, and neuroimaging studies^5–8^ have provided increasingly convincing empirical evidence for the existence of sound symbolism. Given the converging body of evidence that has been published in the field in recent years, the existence of sound symbolism in many different languages and cultures around the world is now incontrovertible^9–12^. Recent studies have also suggested that sound symbolism, or iconicity in general, is advantageous in early language learning^11, 13, 14^.

One of the controversies in the research on sound symbolism concerns the question of which features constitute the main drivers of sound-symbolic effects. While previous studies have mostly focused at phonological features^2, 7, 15–19^, acoustic drivers of sound symbolism (i.e., spectrotemporal features of speech sounds) have received little scholarly attention. Examining acoustic features seems particularly timely as there is no consensus as to which level of analysis (i.e., phonological, articulatory, or acoustic) best accounts for sound-symbolic effects (and many authors indeed do not seem to have a clear hypothesis). In addition, few studies have directly compared different kinds of sound symbolism (such as sound-size and sound-shape symbolism) to test whether they are driven by the same or different acoustic features. Specifically, the extensive literature on sound symbolism has been taken to suggest that the established “large-sounding” phonemes, such as /a/, /o/, /u/, and “small-sounding” phonemes, such as /e/, /i/^20–22^, are also the typical “round-sounding” and “angular-sounding” phonemes, respectively^4, 6, 7, 23–25^. However, fundamental acoustic features of speech sounds may well vary with regards to their sound-symbolic meaning, and thus affect size and shape ratings differently. Assessing the phonetic attributes of sound symbolism also provides a finer picture of what underpins individuals’ biases to link otherwise unrelated sounds and meanings to one another^26^. It has been shown in artificial language learning tasks that non-arbitrary word-meaning associations are easier to learn than arbitrary word-meaning associations^27^, but it remains unclear what exactly the participants are tracking during the perception of the artificial sounds.

In order to address these important gaps in the literature, the present study was designed to determine which acoustic feature(s) best characterise “large/round” sounding phonemes and “small/angular” sounding phonemes. Specifically, we examined whether a set of key acoustic features reliably predicted size and shape symbolism. Our acoustic analyses were focused exclusively on the acoustic features of vowels, because consonants do not have common acoustic parameters; rather, the most important acoustic features of consonants vary across different consonant classes. Vowels are identified by their fundamental frequency f0 and the frequencies of their first three formants (F1-F3) at the vowel center. Higher formants (e.g., F4 or F5) have little influence on vowel distinctiveness, but rather on speaker differentiation, as they are primarily determined by a speaker’s physiology (e.g., head size). As vowels are inherently dynamic, their duration, and dynamic spectral cues (e.g., changes in F1-F3 over time) also affect vowel identification. Vowel quality is also influenced by coarticulation, and a preceding consonant will often modulate the formants of a subsequent vowel (captured in spectrotemporal changes in F1-F3).

Which acoustic vowel features might drive sound-symbolic judgments? Sound-size symbolism should be associated with acoustic features that express size and/or intensity. For instance, among individuals of (mammal) species, greater physical size is commonly associated with a lower fundamental frequency f0 in﻿ vocalizations^28, 29^. f0 also varies across the different speech sounds produced by an individual. To the extent that intra-individual changes in f0 are interpreted similarly to inter-individual changes in terms of their implied size, we therefore predict speech sounds with lower f0 would lead to increased size judgments. The latter should also be affected by formants that indicate greater opening of the oral cavity in an iconic way, as larger opening represents larger size. Therefore, the first formant F1, which increases with lower tongue position and greater jaw opening, should be positively related to visual size judgments. Finally, intensity-related features such as loudness and duration might also affect size judgments. As higher-intensity sounds tend to correspond to larger objects than sounds of lower intensity^28, 30–32^, the intrinsic loudness of vowels should also be associated with the size of an object. Thus, vowels having a higher intensity should correspond to larger objects than vowels with a lower intensity. Similarly, vowels with a longer duration should be associated with larger objects than vowels that are associated with a shorter duration.

Which acoustic vowel properties will have the greatest influence on sound-shape-symbolic judgments, such as judgments of visual roundness versus angularity? Spectral features reflecting lip rounding may influence sound-shape symbolism due to the perceptual analogy between lip rounding and visual roundness. In acoustic terms, lip rounding lengthens the entire vocal tract and therefore lowers all formants, especially F2 and F3. Backing and rounding have reinforcing acoustic effects, as both lower F2^33^. We therefore predict that sounds with a lower F2 and F3 will be associated with more rounded shapes, while sounds with a higher F2 and F3 will be associated with more angular shapes instead.

To compare the relative influence of acoustic parameters on sound-size versus sound-shape ratings, two experiments were conducted. Experiment 1 assessed the role of acoustic features in size-symbolic judgments, while Experiment 2 investigated the role of the same features in shape-symbolic judgments.

## Results

### Experiment 1: Sound-size judgments

In each trial of Experiment 1, the participants heard one of 100 non-word CV (consonant + vowel) sounds via speakers and saw a size scale on the screen (see Methods section). The participants then had to indicate, by pressing a number from one to five on the keyboard, which visual size they thought matched the sound. Each CV sound was presented three times, giving rise to a total of 300 trials.

We used a linear mixed model approach to predict the participants’ responses on the size scale as a function of several acoustic parameters of the CV sounds (Model 1): the standardized values for f0, the formants F1-F3, duration, intensity, and the interaction term of F1 and F2 (see Methods section). Dummy variables controlling for the influence of consonant context of the vowels (i.e., 7 consonant groups: glides, liquids, nasals, voiced fricatives, voiced stops, voiceless fricatives, and voiceless stops) were also included. To account for the increased false discovery rate due to the high number of terms in the model, a Benjamini-Hochberg correction was applied to the resulting raw p-values; all raw p-values reported below remain significant using at least a false discovery rate of 0.15 (or lower).

The model (Model 1, see Table 1) provided partial support for our predictions: Consistent with our prediction that higher F1 and longer vowel duration lead to higher size ratings, increased F1 (b = 0.08, p = 0.047) and duration (b = 0.08, p = 0.007) were indeed associated with larger size. However, contrary to our predictions and to the general findings in the literature^28, 30–32^, neither intensity (p = 0.414), nor f0 (p = 0.186) significantly affected size ratings. The model also revealed several unpredicted effects: Size ratings were negatively related to F2, as lower F2 led to higher size ratings (b = −0.15, p < 0.001). There was also a significant interaction of F1 and F2 (b = −0.07, p = 0.024), such that the positive effect of F1 on size ratings increased as F2 decreased. Finally, size ratings were negatively affected by several of the consonant category dummies (relative to glides). The model accounted for 23.6% of the total variance in size ratings.

**Table 1:** Linear mixed models for Experiments 1 (Model 1) and 2 (Model 2), and for the combined data of Experiments 1 and 2 (Model 3).

	Size data (Model 1)			Shape data (Model 2)			Merged data		
	*B*	*CI*	*p*	*B*	*CI*	*p*	*B*	*CI*	*p*
Fixed Parts									
Intercept	2.99	2.81–3.17	<0.001	3.64	3.41–3.86	<0.001	3.64	3.42–3.85	<0.001
f0	−0.04	−0.11–0.02	0.186	−0.05	−0.14–0.03	0.243	−0.05	−0.13–0.03	0.212
F1	0.08	0.00–0.16	0.047**	−0.06	−0.16–0.05	0.281	−0.06	−0.15–0.04	0.247
F2	−0.15	−0.23–−0.07	<0.001***	−0.19	−0.29–−0.08	<0.001***	−0.19	−0.28–−0.09	<0.001***
F3	−0.01	−0.08–0.05	0.746	0.07	−0.02–0.16	0.121	0.07	−0.01–0.15	0.093
Duration	0.08	0.02–0.13	0.007***	0.04	−0.03–0.12	0.269	0.04	−0.03–0.11	0.229
Intensity	−0.03	−0.11–0.04	0.414	0.02	−0.09–0.13	0.715	0.02	−0.08–0.12	0.691
F1 × F2	−0.07	−0.13–−0.01	0.024***	0.00	−0.08–0.08	0.965	0.00	−0.07–0.07	0.962
Category (baseline: Glide)									
*Liquid*	−0.14	−0.41–0.13	0.300	−0.09	−0.42–0.23	0.584	−0.09	−0.40–0.22	0.566
*Nasal*	−0.29	−0.54–−0.05	0.020***	−0.39	−0.70–−0.08	0.015***	−0.39	−0.68–−0.10	0.009***
*Voiced Fricative*	−0.41	−0.61–−0.21	<0.001***	−0.61	−0.89–−0.33	<0.001***	−0.61	−0.87–−0.36	<0.001***
*Voiced Stop*	−0.66	−0.86–−0.45	<0.001***	−0.57	−0.85–−0.28	<0.001***	−0.57	−0.82–−0.31	<0.001***
*Voiceless Fricative*	−0.78	−1.00–−0.56	<0.001***	−0.31	−0.61–−0.01	0.045**	−0.31	−0.59–−0.04	0.028**
*Voiceless Stop*	−0.86	−1.12–−0.61	<0.001***	−0.83	−1.14–−0.51	<0.001***	−0.83	−1.13–−0.53	<0.001***
Task: Size							−0.65	−0.87–−0.42	<0.001***
Task: Size × f0							0.01	−0.07–0.08	0.861
Task: Size × F1							0.14	0.05–0.23	0.004***
Task: Size × F2							0.04	−0.06–0.13	0.464
Task: Size × F3							−0.08	−0.16–−0.00	0.042*
Task: Size × Intensity							−0.05	−0.15–0.04	0.281
Task: Size × Duration							0.04	−0.03–0.10	0.310
Task: Size × Liquid							−0.05	−0.37–0.27	0.751
Task: Size × Nasal							0.09	−0.19–0.38	0.513
Task: Size × Voiced Fricative							0.20	−0.06–0.46	0.136
Task: Size × Voiced Stop							−0.09	−0.35–0.16	0.476
Task: Size × Voiceless Fricative							−0.47	−0.76–−0.18	0.002***
Task: Size × Voiceless Stop							−0.04	−0.35–0.28	0.820
Task: Size × F1 × F2							−0.07	−0.14–−0.00	0.051*
Random Parts									
σ^2^		0.999			0.996			0.997	
τ_00, Sound_		0.048			0.099			0.062	
τ_00, ID_		0.000			0.000			0.082	
N_Sound_		100			100			100	
N_ID_		30			30			60	
ICC_Sound_		0.030			0.062			0.037	
ICC_ID_		0.000			0.000			0.048	
Observations		9000			9000			18000	
R^2^/Ω_0_^2^		0.236/0.233			0.252/0.249			0.309/0.308	

All continuous predictors standardized. p-values are raw p-values without corrections for multiple testing. Asterisks indicate significant effects according to the Benjamini-Hochberg correction for multiple testing, for different false discovery rates (FDR: *** = 0.05, ** = 0.10, * = 0.15).

### Experiment 2: Sound-shape judgments

The procedure of Experiment 2 was exactly the same as Experiment 1, except that the participants were presented with a shape scale on the screen and had to indicate, on a scale of one to five, which visual shape (more rounded versus more angular) matched the CV sound they heard (see Methods section). Each CV sound was presented three times, giving rise to a total of 300 trials.

We used a linear mixed model (Model 2) to predict the participants’ responses on the shape scale as a function of the same acoustic parameters as in Model 1. The same Benjamini-Hochberg correction was applied to the raw p-values to correct for the increased false -discovery rate, and all corrected p-values remained significant at a false discovery rate of 0.15 (or lower).

The model (Model 2, see Table 1) provided partial support for our prediction that formants linked to lip rounding would be associated with shape ratings. Specifically, F2 significantly affected shape ratings (b = −0.19, p < 0.001), such that sounds with higher F2 (less lip rounding) were perceived as more angular. However, F3 did not predict roundness ratings (p = 0.121). Shape ratings were also negatively related to several consonant categories (relative to glides). None of the other effects reached significance. The model accounted for 25.2% of the total variance in shape ratings.

### Pooled datasets from Experiments 1 and 2

After analyzing the data from Experiments 1 and 2 separately, the datasets were merged for a more formal comparison of the role of the tested predictors across the size- and the shape-rating tasks. Such a pooled analysis does not only provide increased statistical power relative to separate analyses, but is also necessary from a statistical perspective to establish the relative impact of acoustic predictors in both rating tasks. It involves assessing the interactive effects of a new variable “task type” (a dummy that classifies each trial based on its source experiment) and the various acoustic parameters in the merged dataset. In other words, the interaction terms between task type and the acoustic parameters provide direct statistical tests of the relative influence of the predictors across size versus shape ratings (see Methods section for additional details on this approach).

Using the merged dataset, we ran an additional linear mixed model that included interaction terms between (a) a new dummy variable encoding sound-size ratings versus sound-shape ratings and (b) the various acoustic features in order to obtain direct statistical tests of the relative influence of the predictors in size versus shape ratings (see Methods section). All raw p-values reported below survived a Benjamini-Hochberg correction at a false discovery rate of 0.15 (or lower).

**Figure 1:**
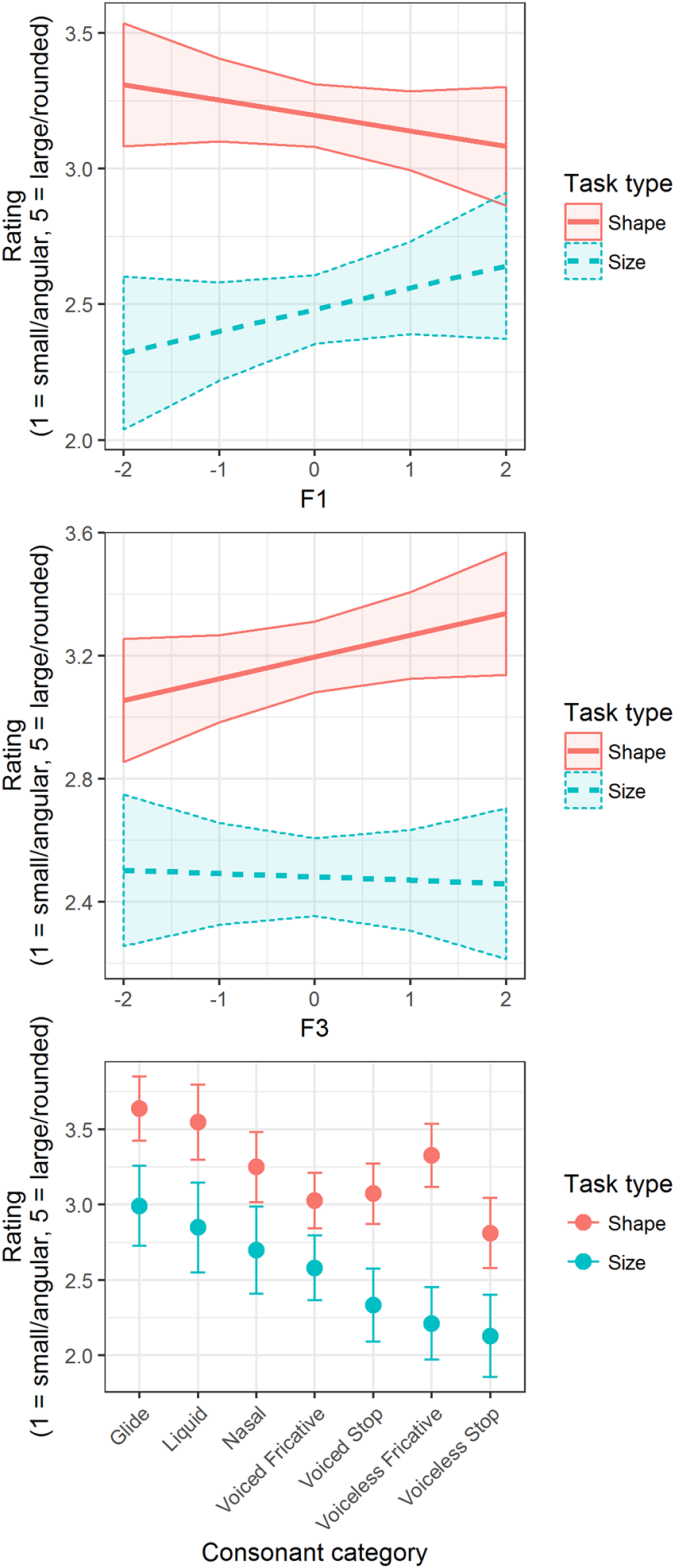
Fitted values for the interactive effects of task type and standardized formants F1 (top panel) and F3 (middle panel), and consonant category (bottom panel) on participants’ size and shape ratings, based on the pooled data from Experiments 1 and 2 (Model 3). The shaded bands represent 95% confidence bands, error bars represent 95% confidence intervals. Higher F1 leads to higher size ratings, but has little effect on roundness ratings (top). Higher F3 leads to higher ratings of roundness, but not size (middle). Size and shape ratings also are differently affected by several of the consonant groups (bottom).

**Figure 2:**
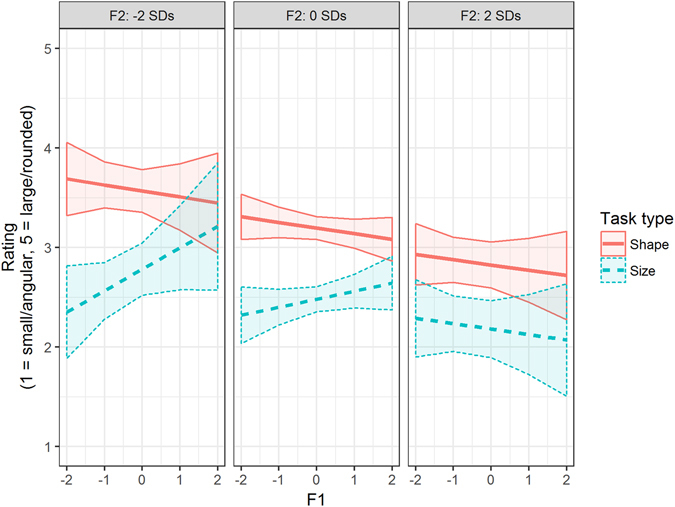
Fitted values for the three-way interactive effect of task type and standardized formants F1 and F2 on participants’ size and shape ratings, based on the pooled data from Experiments 1 and 2 (Model 3). Shaded bands represent 95% confidence bands. The effect of F1 is moderated by F2 in size ratings, but not by shape ratings. Consequentially, size ratings peak at combinations of high F1 and low F2, whereas roundness ratings are highest for low F2, regardless of F1.

The model (Model 3, for a summary, see Table 1) revealed significant main effects for F2 (b = −0.19, p < 0.001), task type size (b = −0.65, p < 0.001), and several of the consonant groups. There were several significant two-way interactions between task type and F1 (b = 0.14, p < 0.004), task type and F3 (b = −0.08, p < 0.042), and task type and one of the consonant groups. These two-way interactions, depicted in Fig. 1, suggest that F1 positively affects size ratings and has little effect on shape ratings, while F3 positively affects shape (i.e., roundness) ratings and has little effect on size ratings. There was also a marginally significant three-way interaction between task type, F1, and F2 (b = −0.07, p < 0.051). The pattern of this interaction, depicted in Fig. 2, suggests that shape ratings were mainly driven by F2, while size ratings were driven by a combination of F1 and F2 (consistent with the significant F1 × F2 interaction in Experiment 1). Specifically, shape ratings were highest (i.e., most round) for sounds with lower F2, whereas size ratings were highest for sounds with higher F1 and lower F2 (e.g., /a, o/). The model accounted for 30.9% of the total variance in size and shape ratings.

Finally, a Pearson correlation coefficient was calculated in order to assess the relationship between the averaged size judgments (Experiment 1) and the average roundness judgments (Experiment 2) for the 100 CV sounds. The analysis revealed a significant correlation (*r*(98) = 0.61, *p* < 0.001), indicating that, on average, those CV sounds that were perceived to be larger were also perceived to be more rounded.

## Discussion

The present study is the first to examine the acoustic drivers of two distinct types of sound symbolism—specifically size symbolism and shape symbolism—within the same experimental setup. The results of Experiments 1 and 2 revealed different patterns of sound-symbolic mappings for size and shape symbolism. Size symbolism, on the one hand, was influenced by formants F1 and F2 and particularly their interaction, and also by duration. Visual size judgments in Experiment 1 were higher for sounds with a higher F1 and simultaneously a lower F2 and for sounds with longer duration. However, we did not find significant main effects of f0 and intensity on size judgments. These null effects might be attributable to a lack of variance in f0 and intensity in the used CV stimuli: As we were interested in vowel-intrinsic intensity and f0, we kept the recording volume constant at around 70 dB for all CV sounds; and the speaker who recorded the 100 CV sounds did not vary her voice pitch and loudness much during recording. Future experiments with stimuli specifically designed to vary in intensity and f0 may shed more light on the relative influence of these parameters on size rating. Shape symbolism, on the other hand, was predicted by different acoustic features of vowels. Specifically, shape judgments in Experiment 2 were influenced by F2, such that visual roundness judgments decreased when F2 increased. F3, which reflects lip rounding, did not have a clear effect on shape ratings in Experiment 2. However, the subsequent pooled analysis of Experiments 1 and 2 revealed that F3 is more influential in shape ratings, with higher values of F3 resulting in higher roundness perception. These findings provide support for our prediction that F2 and F3 influence shape ratings due to their association with vowel frontness and lip rounding.

Extending previous studies on sound symbolism, our findings suggest that sound-symbolic ratings are not merely based on broad categorical contrasts, such as round/unround or front/back vowels. Instead, individuals seem to base sound-symbolic judgments on detailed acoustic cues extracted from speech sounds, which vary across judgment dimensions. We also provide a more nuanced picture for the widespread assumption that the established “large-sounding” (e.g., /a/, /o/, /u/) and “small-sounding” phonemes (e.g., /e/, /i/)^20–22^ are congruous with the typical “round-sounding” and “angular-sounding” phonemes, respectively^4, 6, 7, 23^: Size and shape symbolism, although driven by different sets of acoustic features, share one common cue (i.e., F2). Thus F2, which increases with vowel frontness, leads to the significant correlation between the average size and roundness scores.

Our study represents a first step to experimentally testing the role of different acoustic cues in different cross-modal correspondences, which has long been suggested in descriptive linguistics^34–36^. However, the set of acoustic parameters tested in the present study was by no means exhaustive. While we examined some of the most prominent features of vowels, it is important to note that additional acoustic parameters may play an important role in differentiating sound-symbolic judgements (implied also by the moderately high pseudo R-squareds in our models). Similarly, acoustic features of consonants were not considered in the present study, because different consonant categories are often identified by different sets of acoustic features, which renders their combined analysis difficult. Future studies could therefore fruitfully explore the impact of additional acoustic parameters.

The finding that size rating is positively related to F1, which reflects the progressive opening of the jaw, is consistent with the music protolanguage hypothesis^37^, first proposed by Charles Darwin^38^. It holds that speech sounds originated in the imitation and modification of available environmental sounds. If this hypothesis is true, we should expect to observe iconic relationships between acoustic properties of speech sounds and information about the size, proximity, and degree of energy of a sound source in the environment^39^. For example, larger species tend to produce sounds with lower F1 and longer duration than smaller animals, so we would use sounds with lower F1 and longer duration to represent large-sized objects. Indeed, we found that longer duration and lower F1 are positively related to people’s size ratings.

Motivated connections between words and meanings have also been proposed to benefit language learnability^27^. Our study suggests that a “motivation” can consist of a set of acoustic features, and that different dimensions of sound-meaning associations are expressed through different acoustic cues. From a learning perspective, it would facilitate disambiguation if different visual features were mapped to different acoustic parameters. In terms of the lexicon, we suggest that sound and meaning may be mapped not at the word level, but at the finer acoustic level. Therefore, although sound-symbolic words only make up a small portion of the lexicon^36^ such as idiophones, sound-symbolic acoustic features could be pervasive, and influence word meaning at the more implicit acoustic level.

The results of the present study also have important implications for the “continuous-contrastive marking” problem^12, 40^, namely, the question whether sound-symbolic effects are continuous (i.e., the “large-”, “medium-” and “small-sounding” phonemes correspond to “large”, “medium”, and “small” sizes respectively) or contrastive where only two semantically contrastive meanings (e.g., large-small, round-angular, low-high) are marked with phonetically contrastive sounds (e.g., the low back vowel /a/ and the front high vowel /i/). The “continuous-contrastive marking” problem is directly related to the debate on the evolution of sound symbolism in natural language^12^. Two explanations dominate this debate: The “crossmodal account” holds that sound symbolism has evolved because speakers match gestural or auditory parameters of speech sounds to physical properties in the visual or other modalities. Both speech gestures and auditory parameters can be graded to express specific magnitudes of a referent property (e.g., medium size, medium angularity). In contrast, a “statistical account” posits that sound symbolism has evolved merely as a consequence of speakers observing randomly evolved patterns in the phoneme distribution of a language. For instance, back sounds may have become more prominent in words that describe large objects simply by chance—without any inherent connections between speech sound and referent. As argued by Thompson and Estes^12^, this latter, statistical account can only explain sound-symbolic marking of two contrastive values of the given physical property, such as “small” and “large”, since the observed statistical associations are based on semantic contrasts. The crossmodal explanation, however, accommodates sound-symbolic marking of continuous degrees of the given physical property, such as “small”, “medium”, and “large”, since both gestures and sound properties are continuous. For example, a mid-sized object could be indicated by a moderate mouth gesture and mid-range loudness.

Thompson and Estes^12^ tested whether the size of an object (small, medium, or large in size) would linearly predict the number of “small-sounding” and “large-sounding” phonemes in their names, and their results revealed that as the size of the objects increased linearly, so too did the number of large-sounding phonemes in their preferred names. The present study extends and generalizes the findings of Thompson and Estes^12^ from sound-size to sound-shape symbolism, and from number of phonemes to acoustic properties. We showed that size-symbolic judgments varied linearly as a function of F1, F2, and duration, and shape-symbolic judgments varied linearly as a function of F2 and F3. Taken together, these results indicate that continuous marking may apply to auditory-visual feature mapping in general (such as F1-size, F2-shape, etc.), and hence support the “cross-modal” account that links auditory and visual modalities in a continuous manner.

In terms of potential applications, the present findings also have implications for the field of consumer psychology. Research in this field has shown that verbal stimuli (such as brand names) can have sound-symbolic effects on consumers’ perceptions and decision-making^16, 17, 41–50^. These studies have mostly varied a very limited number of (mostly high-level) speech parameters (e.g., front vs. back phonemes) and are typically based on relatively broad, contrasting categories of sound-symbolic meaning (e.g., front vowels have been characterized as smaller, lighter, milder, etc. than back vowels^16^). The present findings suggest that, depending on the intended sound-symbolic effect (e.g., communicating specific product shapes vs. communicating information about their sizes), different acoustic parameters will have the largest impact. For instance, larger size can be most effectively expressed by using vowels with high F1, low F2, and longer duration. By contrast, visual roundness is most effectively communicated by vowels that are low in F2 and high in F3.

In sum, the present study suggests that different acoustic drivers underlie size and shape symbolism. Specifically, F1 in combination with F2, and duration predicted size symbolism, while shape symbolism was associated with F2 and F3. These findings portray sound symbolism as a process that is not merely based on categorical contrasts, such as the differentiation between round and unround or front and back vowels. Instead, individuals base their sound-symbolic judgments on specific sets of acoustic cues extracted from the sounds, which vary across judgment dimensions.

Further research could extend the comparative methodology of the present study to additional sound-meaning correspondences, such as sound and weight, taste, or emotion, and extend the comparisons to children of different age groups. Tracking the development of different types of sound symbolism and cross-modal correspondences is crucial for our understanding of the nature versus nurture debate on sound symbolism. Studies using more implicit methods, such as artificial learning tasks^51, 52^, may reveal more about the role of sound-symbolic effects in natural language learning and processing. There are also scant neuroimaging studies on the topic sound symbolism, with those that have been published mainly focusing on the existence of sound-symbolic effects in adults^8^ and children^13^. An EEG study with children confirmed increased processing demands in sound-meaning mismatch conditions^13^, and an fMRI study with adults located the left superior parietal cortex as the potential site for sound-symbolic mapping^8^. Extending this existing literature with the current results in mind, we suggest further neuroimaging studies that specifically compare the brain activation patterns for different types of sound-symbolic judgments to help uncover the neural basis underlying different types of sound symbolism.

## Methods

### Participants

Thirty Oxford University students took part in Experiment 1 (19 female, mean age = 22.3 years). Seven of the participants were bilingual English speakers, and the remainder were native English speakers. A new sample of thirty Oxford University students (21 female, mean age = 21.4 years) took part in Experiment 2. Six of the participants were bilingual. All participants had normal or corrected-to-normal vision.

Both Experiments 1 and 2 lasted for 30–40 minutes each and were approved by the Oxford University ethics committee. The methods were carried out in accordance with the “policy on the ethical conduct of research involving human participants and personal data” of the University of Oxford. The participants gave written informed consent before taking part in the experiments, and were reimbursed £10 for their participation.

### Visual stimuli

**Figure 3:**
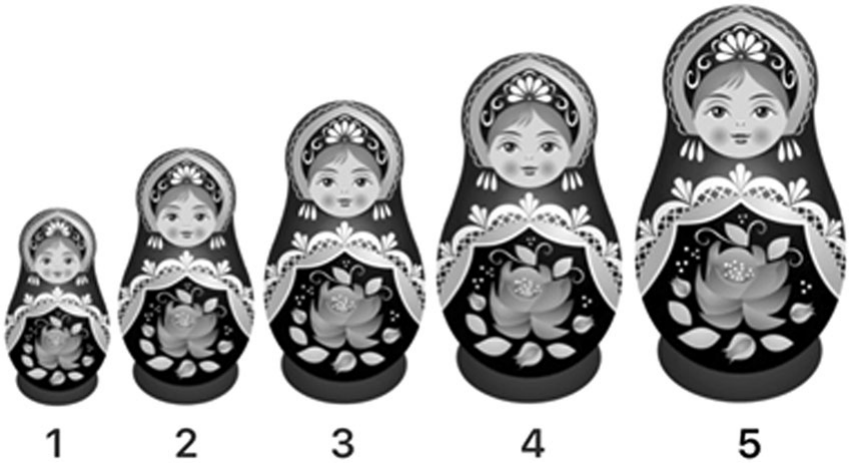
One of the visual stimuli used in Experiment 1. Original images were purchased from © Elmmksat at Dreamstime.com.

The visual stimuli for Experiment 1 consisted of greyscale pictures of Russian dolls varying in size. The Russian dolls were chosen as the visual stimuli because they intuitively convey the meaning of differing sizes (i.e., smaller dolls can be fitted inside larger dolls). As a consequence, there is no need for an extra reference object, as used by Thompson and Estes^12^, to indicate that what varies is the size of the object (rather than, say, the distance of the object). 5-point scales were constructed from sets of five dolls that varied in size. The five dolls (from left to right) subtended 1.7 cm × 3.2 cm, 2.2 cm × 4 cm, 2.6 cm × 4.8 cm, 3 cm × 5.5 cm and 3.4 cm × 6.3 cm at a viewing distance of 40 cm (aligned horizontally; see Fig. 3 for an example). In order to minimize a potential confounding influence of the particular pattern on the body of the dolls, five different versions of the visual stimuli were created using dolls with five different patterns (see Supplementary Fig. S1 for an illustration of the different patterns).

**Figure 4:**
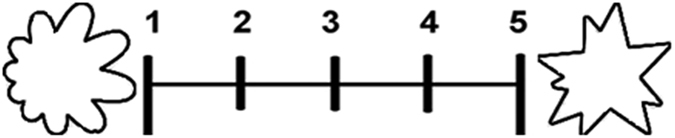
One of the visual stimuli used in Experiment 2.

The visual stimuli for Experiment 2 included five pairs of round and angular shapes (see Supplementary Fig. S2 for all shapes). The shapes were all black and white, generated by the online pattern generator “subblue” (http://www.subblue.com/projects; downloaded on 04/05/2012), and all fit into a square subtending 3 cm × 3 cm^5^. The shapes were shown at the ends of a 5-point scale (aligned horizontally with a separation of 9.82 cm on the screen; see Fig. 4 for an example).

### Auditory stimuli

The auditory stimuli for both Experiments 1 and 2 included 100 CV non-words (20 consonants x 5 vowels; see Table 2). These 100 CV sounds were selected because they include a wide spectrum of acoustic features and present vowels in different consonant contexts. Using different consonant contexts to test the effects of vowel features is important as consonant context may affect the formant patterns of adjacent vowels^53^. By testing vowels in different consonant environments, we thus reduce the likelihood that any obtained effects of vowel features may depend on the presence of specific consonants, which increases the generalizability of our findings. Further, presenting vowels in different consonant contexts helped us to reduce participant fatigue during the experiments. The sounds were recorded as 16-bit stereo files at a sampling frequency of 44,100 Hz by a female phonologist, who was a native English speaker. The stimuli can be downloaded at osf.io/xsejv.

**Table 2:** The auditory stimuli utilized in Experiments 1 and 2.

/wa/	/ja/	/la/	/ra/	/ma/	/na/	/za/	/va/	/ʒa/	/ða/
/wu/	/ju/	/lu/	/ru/	/mu/	/nu/	/zu/	/vu/	/ʒu/	/ðu/
/wo/	/jo/	/lo/	/ro/	/mo/	/no/	/zo/	/vo/	/ʒo/	/ðo/
/we/	/je/	/le/	/re/	/me/	/ne/	/ze/	/ve/	/ʒe/	/ðe/
/wi/	/ji/	/li/	/ri/	/mi/	/ni/	/zi/	/vi/	/ʒi/	/ði/
/ba/	/da/	/ga/	/sa/	/fa/	/∫a/	/θa/	/pa/	/ta/	/ka/
/bu/	/du/	/gu/	/su/	/fu/	/∫u/	/θu/	/pu/	/tu/	/ku/
/bo/	/do/	/go/	/so/	/fo/	/∫o/	/θo/	/po/	/to/	/ko/
/be/	/de/	/ge/	/se/	/fe/	/∫e/	/θe/	/pe/	/te/	/ke/
/bi/	/di/	/gi/	/si/	/fi/	/∫i/	/θi/	/pi/	/ti/	/ki/

### Acoustic measurements

Several acoustic properties of the auditory stimuli were measured using Praat (www.praat.org): We first segmented the consonant and the vowel of the 100 CV sounds, then we measured f0 and formants F1-F3 in the middle of the vowel, peak intensity, and duration of the vowel. Our acoustic analyses were focused exclusively on the acoustic features of vowels, because consonants do not have common acoustic parameters; rather, the most important acoustic features of consonants vary across different consonant classes.

### Procedure

The participants were seated in front of a 17-inch screen in a silent testing room. The centre of the screen was positioned at the participants’ eye-level at a distance of 40 cm. The volume of all sounds was kept in a comfortable listening range around 70 dB(A).

In Experiment 1, the participants heard a CV sound and saw a size scale on the screen (the visual ornament pattern of the dolls was randomized per trial). The participants then had to indicate, by pressing a number from one to five on the keyboard, which size of doll they thought matched the sound. Each sound was presented three times, giving rise to a total of 300 trials. The presentation of the auditory stimuli was completely randomized, but the orientation of the scales was preserved (small to large from left to right) across trials. After the experiment, the participants were debriefed; none of them reported previous knowledge of phonetic symbolism.

In Experiment 2, the participants heard a CV sound and saw a shape scale on the screen. They then had to indicate, by pressing a number from one to five on the keyboard, the degree of roundness that best matched the sound. Each sound was presented three times, giving rise to a total of 300 trials. The presentation of the stimuli was randomized, as was the orientation of the scales.

### Data analysis

To analyze the impact of acoustic features on size ratings (Experiment 1) and shape ratings (Experiment 2), we used linear mixed-effects regression using the function *lmer*() from the *lme4* package in R version 3.3.1^54^. Linear mixed model regression is now considered the standard for analysing repeated-measures data in psycholinguistic experiments, and provides several important advantages over traditional methods such as repeated-measures ANOVA^55^. We specified two separate models for Experiment 1 and Experiment 2. The models predicted the participants’ responses on the size (Model 1) and shape (Model 2) scales, respectively, as a function of the values for f0, F1-F3, duration, intensity, and the interaction term of F1 and F2. We included the interaction between F1 and F2 because differences in vowel quality are well described by changes in the first two formant frequencies. A control variable that encoded all speech sounds into seven different consonant categories (glides, liquids, nasals, voiced fricatives, voiced stops, voiceless fricatives, voiceless stops) was also included as a fixed effect to explain additional variance (adding six dummy variables to the models). In line with suggestions by Barr and colleagues^56^, the models included the maximal random effects structure justified by the design; that is, random intercepts for both participants and stimuli and random by-participant slopes for each of the acoustic predictors as well as for the interaction of F1 and F2 (but no random by-stimulus slopes, since acoustic predictors did not vary within stimuli). These “maximal” models did not converge. We thus followed guidelines by Barr *et al*.^56^ on model non-convergence and removed correlations between the random effects. As the resulting models still did not converge, and in order to reduce multicollinearity induced by the interaction terms in the model, all predictors for the acoustic features were standardized. The resulting models converged. After standardizing, none of the VIF scores was greater than 2.16 (Experiment 1) and 2.39 (Experiment 2), indicating that multicollinearity was not a problem^57^.

p-values for Models 1 and 2 were calculated using the *sjt*.*lmer*() function from the *sjPlot* package with a Kenward-Roger approximation of degrees of freedom^58^. While an argument can be made that the model includes planned tests and therefore requires no correction for multiple testing, we applied a Benjamini-Hochberg correction controlling the raw p-values at three different false discovery rates (FDR: 0.05, 0.10, 0.15)^59^ to further protect against an increased false discovery rate (see Table 1).

The data obtained in Experiments 1 and 2 provide valuable insights into the effects of acoustic properties on sound-size and sound-shape ratings, respectively. However, analyzing these data separately does not allow us to statistically compare the effects of acoustic properties across the two types of phonetic symbolism. That is, separate analyses do not provide direct statistical tests to assess whether the effect of any given acoustic parameter differs across sound-size and sound-shape ratings—which is the main goal of the present research. A formal comparison of the role of the tested predictors across the size and shape rating tasks requires pooling the data from Experiments 1 and 2 and testing the interactive effects of task type and the acoustic parameters. We thus merged the datasets and added a new variable called “task type” that coded trials as either “size rating” or “shape rating” trials, depending on the source dataset. The interaction terms between this new variable and the various acoustic features provide direct statistical tests of the relative influence of the predictors across size versus shape ratings. From a methodological point of view, this pooling seems appropriate, as the data for both experiments were collected under virtually identical circumstances (e.g., in terms of time and place of data collection, lab setup, procedure). One potential limitation of our design stems from the fact that the size and shape rating experiments used different rating scale formats. Size ratings were done on a scale in which each value was represented by a doll image, while shape ratings were done on a scale where only the anchors (angular vs. rounded) were represented by abstract line drawings. This implies that task type is confounded with rating scale format. However, this confounding would mainly affect the interpretation of any main effect of task type, while the focus of this analysis are potential interactions between task type and the acoustic predictors. Regarding these interactions, it is not obvious how varying effects of acoustic parameters across the two task types could be explained away by variations in the scale format, and we therefore proceeded with our analyses. The resulting pooled dataset contained 18,000 trials (60 participants, 300 trials each).

A linear mixed model (Mo﻿de﻿l 3) was used to predict the participants’ response on the size or shape scale (5-point) as a function of task type (dummy-coded, size vs. shape rating), standardized values for f0, F1-F3, duration, intensity, the interaction terms of task type and the acoustic features, an interaction term of F1 and F2, and the three-way interaction between task type, F1, and F2. A control variable coding seven different consonant categories (glides, liquids, nasals, voiced fricatives, voiced stops, voiceless fricatives, and voiceless stops) was also included as a fixed effect to explain additional variance (adding six dummy variables and their interactions terms with task type to the model). The model included the maximal random effects structure justified by the design; that is, random intercepts for both participants and stimuli, random by-participant slopes for each of the acoustic predictors as well as for the interaction of F1 and F2 (but no random by-stimulus slopes, since acoustic predictors did not vary within stimuli), and a random by-stimulus slope for task type. This “maximal” model did not converge. We thus removed correlations between the random effects. As the resulting model still did not converge, and in order to reduce multicollinearity induced by the interaction terms in the model, all predictors for the acoustic features were standardized. The resulting model converged. After standardizing, none of the VIF scores was greater than 2.41, indicating that multicollinearity was not a problem^57^. p-values for Model 3 were calculated as in Models 1 and 2, and a Benjamini-Hochberg correction was applied.

## Electronic supplementary material

Supplementary Information
