# Supplementary material for: What drives sound symbolism? Different acoustic cues underlie sound-size and sound-shape mappings
Source: Sci Rep. 2017 Jul 17;7:5562. doi: 10.1038/s41598-017-05965-y (PMC5514121; doi:10.1038/s41598-017-05965-y)

What drives sound symbolism?

Different acoustic cues underlie sound-size and sound-shape mappings

Klemens Knoeferle[1*], Jixing Li[2*], Emanuela Maggioni[3], & Charles Spence[4]

[1] Department of Marketing, BI Norwegian Business School, Oslo, Norway

[2] Department of Linguistics, Cornell University, Ithaca, United States

[3] School of Engineering and Informatics, University of Sussex, United Kingdom

[4] Department of Experimental Psychology, University of Oxford, Oxford, United Kingdom

Correspondence concerning this article should be directed to: Klemens Knoeferle, Department of Marketing, BI Norwegian Business School, Nydalsveien 37, 0484 Oslo, Norway, klemens.knoferle@bi.no, +47 464 10 932; Jixing Li, Department of Linguistics, Cornell University, 203 Morrill Hall, Cornell University, Ithaca, New York, USA, jl2939@cornell.edu, +1 607 379 4848

*These authors contributed equally to this work.

Supplementary Information

Figure S1: The five doll patterns used in Experiment 1. Original images were purchased from © Elmmksat at Dreamstime.com.

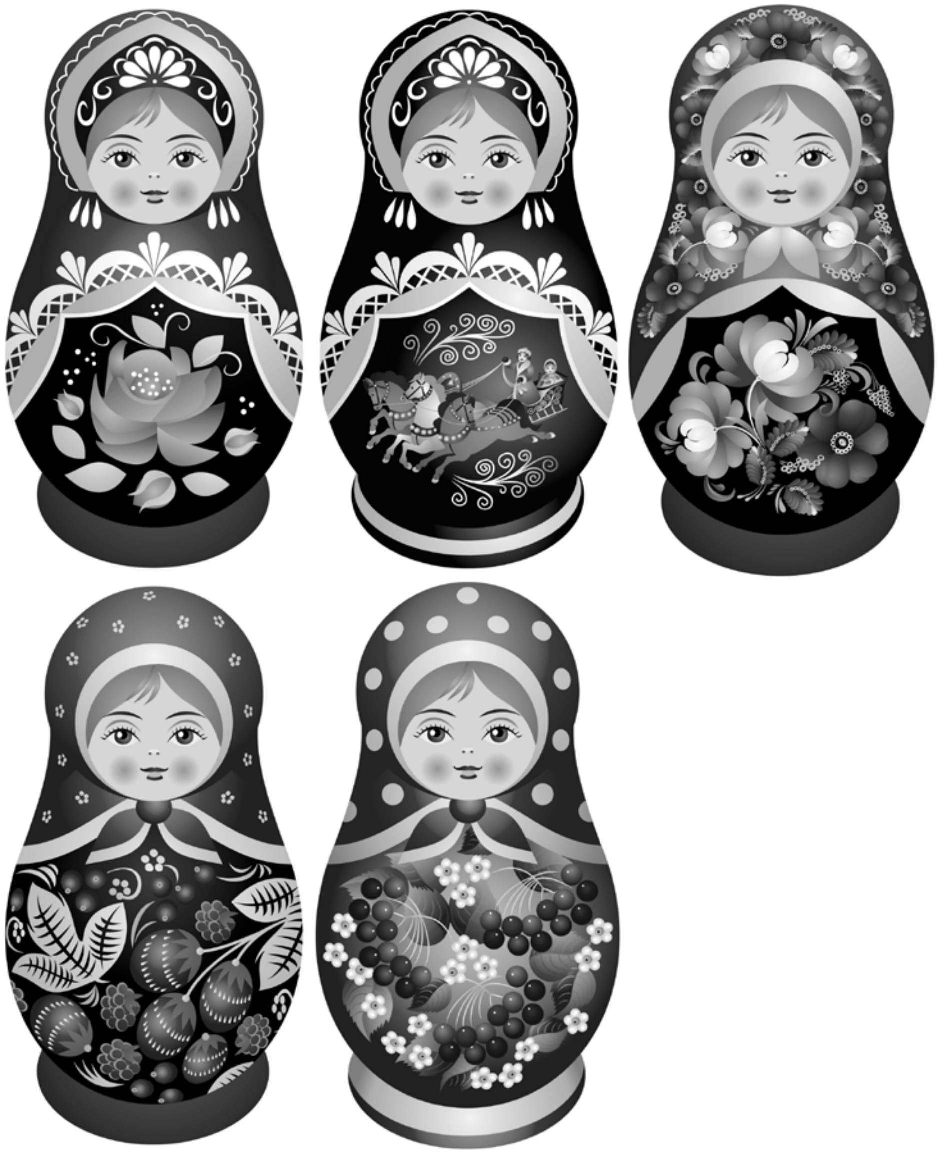

Figure S2: The 10 shapes used in Experiment 2.

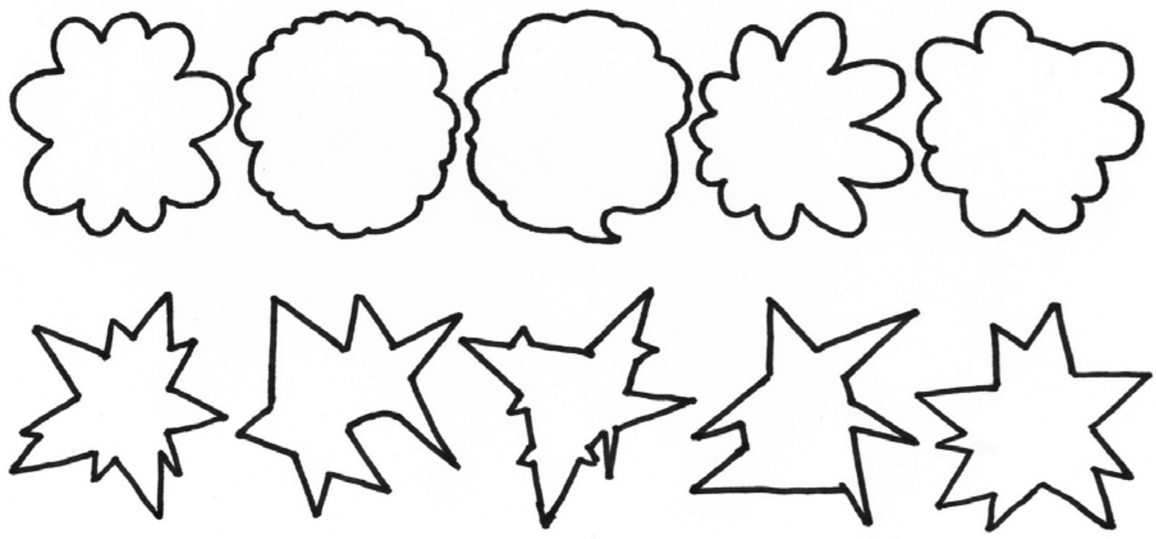

Supplement: Supplementary file 1 — Supplementary Information [file 41598_2017_5965_MOESM1_ESM.pdf]
